# Diagnostic Performance between Histidine-Rich Protein 2 (HRP-2), a Rapid Malaria Diagnostic Test and Microscopic-Based Staining Techniques for Diagnosis of Malaria

**DOI:** 10.1155/2020/5410263

**Published:** 2020-03-27

**Authors:** Jean Baptiste Niyibizi, Emmanuel Kamana Gatera

**Affiliations:** ^1^Department of Medical Laboratory Sciences, Mount Kenya University, Kigali Campus, Kigali, Rwanda; ^2^University of Global Health Equity, Basic Medical Sciences, Butaro-Kigali, Kigali, Rwanda; ^3^JHPIEGO, Rwanda

## Abstract

Malaria presents a diagnostic challenge in most tropical countries such as Rwanda. Microscopy remains the gold standard for diagnosing malaria, but it is labor intensive and depends upon the skill of the examiner. Malaria rapid diagnostic tests (RDTs) have been developed as an easy, convenient alternative to microscopy. This cross-sectional study was conducted at Rukara Health Center which is located in Eastern Province, Kayonza district, Rwanda. One hundred and fifty suspected cases of malaria, who attended Rukara Health Centre, during the period, from 21^st^ June to 30th July 2018, were included in this study. HRP-2 RDTs (CareStart™ Malaria HRP-2 (Access Bio, Inc., Somerset, New Jersey, USA)), for malaria were performed. Thick smears were prepared and Giemsa-stained as recommended; then slides were observed under microscopy and reported quantitatively; RDTs were reported qualitatively (positive or negative). Both RDTs and thick smear results were recorded on data collection sheet. This study included a total of 150 study participants, 87 (58%) females and 63 (42%) males. The patients included in the study did not receive any antimalarial drug. The mean age of the study participants was 31.6 ± 12.4 with the majority of participants being between 25 and 44 years and the minority being above 65 years. The sensitivity of RDT (HRP-2) was calculated and found to be 95.0%, whereas the sensitivity of Giemsa microscopy was 100%. The specificity of RDT (HRP-2) was calculated and found to be 59.2%, whereas the specificity of Giemsa microscopy was 100%. Negative and positive predictive values of RDT are 85.4% and 82.7%, respectively. Negative and positive predictive values of Giemsa microscopy were both 100%. According to the results of the current study, the sensitivity, specificity, and both positive and negative predictive values of Giemsa microscopy are higher than those of histidine-rich protein 2-based rapid diagnostic test for malaria. The results obtained in histidine-rich protein 2-based rapid diagnostic test for malaria parasites should be confirmed with tests with high specificity. Further studies should determine the most appropriate type of rapid diagnostic test of malaria diagnosis to be used in combination with Giemsa microscopy. In addition, sensitivity and specificity of RDT (HRP-2) and Giemsa microscopy should be assessed against molecular biology techniques.

## 1. Introduction

### 1.1. Background of the Study

Malaria is one of the highest killer diseases affecting most tropical countries, especially African countries. It affects over 500 million people worldwide and over one million children die annually from malaria [1]. Of all the human malaria parasites, *Plasmodium falciparum* (*P. falciparum*) is the most pathogenic and is frequently fatal if untreated in time [2]. Traditional practice for outpatients has been to treat presumptively for malaria based on a history of fever, but a significant proportion of those treated may not have parasites (over 50% in many settings) and hence waste a considerable amount of drugs [3]. This old clinical based practice is still relevant today, especially in infants where time spent on getting a confirmatory laboratory diagnosis could lead to increased fatality.

The WHO makes the tentative recommendation that parasite-based diagnosis should be used in all suspected cases of malaria with the possible exception of children in high-prevalence areas and certain other situations [1]. For this recommendation to be adhered to, obviously, rapid and accurate laboratory findings or demonstration of malaria parasite should be established. The traditional method of microscopic identification of parasite, however, is not only daunting in poor power setting but also time consuming and requiring a lot of expertise/training. Thus, microscopy in Africa is generally limited to larger clinics/tertiary centers. This conventional staining of peripheral blood smears/microscopy, however, still remains the gold standard in laboratory diagnosis of malaria [4].

RDTs are commercially available in kit forms with all necessary reagents and the ease of performance of the procedures does not require extensive training or equipments to perform or to interpret the results, and results are read in 12–15 min. RDTs mainly come in two forms. One is antigen-based and normally requires the use of haemolyzed red blood cells while the other is antibody-based and normally requires the use of extracted serum. Generally speaking, antibodies are better expressed in serum otherwise plasma could also stand in place of serum for the antibody-based method [5]. This study correlated the two methods, microscopy and RDTs in the diagnosis of malaria at Rukara Health Center.

Malaria presents a diagnostic challenge in most tropical countries including Rwanda. Microscopy remains the gold standard for diagnosing malaria, but it is labor-intensive and depends upon the skill of the examiner [6]. Malaria rapid diagnostic tests (RDTs) have been developed as an easy, convenient alternative to microscopy, a high-degree of disease spectrum for quick intervention in order to avert danger associated with delayed diagnosis [4]. Widespread prescription of chloroquine in last 10 years in Rwanda to patients not having malaria has been tolerated, partly because chloroquine was so cheap. However, now, artemisinin-based combination therapy (ACT) costs at least 10 times more per treatment. Rapid diagnostic tests (RDTs) for malaria could be considered for most patients in endemic regions, especially in poor power settings where there is shortage of qualified manpower in Africa. However, there is very little evidence, especially from malaria-endemic areas to guide decision-makers on the sensitivity and specificity of these RDTs. Therefore, this study comparatively evaluated the diagnostic performance between rapid malaria diagnostic tests and microscopic-based stain techniques for the diagnosis of malaria in Rukara Health Center.

### 1.2. Objectives of the Study

#### 1.2.1. General Objective

To determine the diagnostic performance between rapid malaria diagnostic test and microscopic-based stain techniques for diagnosis of malaria at Rukara Health Center.

#### 1.2.2. Specific Objectives

- To determine the sensitivity of rapid malaria diagnostic test and microscopic-based stain techniques for diagnosis of malaria.
- To determine the specificity of rapid malaria diagnostic test and microscopic-based stain techniques for diagnosis of malaria.
- To determine the positive and negative predictive values of rapid malaria diagnostic test and microscopic-based stain techniques for diagnosis of malaria.

## 2. Methodology

### 2.1. Research Design

This study was conducted at Rukara Health Center. It is located at Kayonza district in Eastern Province, Rwanda. A cross-sectional study design was used in this study. Target population of this study are all suspected cases of malaria, from various sectors of Kayonza district, Eastern Province, Rwanda, who attended Rukara Health Center during the period from 21^st^ June to 30^th^ July 2017. All patients who are not suspected of malaria diseases were excluded from this study.

### 2.2. Sample Size

The sample size was estimated by using

(1) $$\begin{array}{c} n=\frac{z^{2}p\left(1-p\right)}{d^{2}}, \end{array}$$

where, *n* = required sample size. *z* = confidence level 95% (standard value of 1.96). *p* = estimated prevalence of malaria, we will take 11% obtained as the prevalence of malaria in Eastern Province (Rwanda Health Survey, 2016). *d* = margin of error at 5 % (standard value is 0.05).

Sample size calculation is as follows:

(2) $$\begin{array}{c} n=\frac{1.962\times 0.11\times \left(1-0.89\right)}{0.052}=150.03. \end{array}$$

Finally, the sample size was 264 patients.

### 2.3. Sampling Techniques

A convenience sampling with consecutive design was used to select the research subjects of this study.

### 2.4. Data Collection Techniques

In this study, the demographic data were collected from patient file to data collection form. These were filled with a study ID, demographic (gender and district), and malaria status on microscopy as well as RDTs. The collected data were checked for completeness, edited into Microsoft Excel 2010 sheet, and then imported into IBM SPSS for statistical analysis.

### 2.5. Specimen Collection Procedures

Patient specimens (blood capillary) were used in RDTs for the diagnosis of malaria. Thick smears were prepared and Giemsa-stained as recommended [7]. Giemsa-stained smears were observed under the microscope and reported qualitatively (positive). Giemsa-stained smears were also reported quantitatively using the following formula: parasites/*μ*L blood = number of parasites counted x 8000 white blood cells/*μ*L divided by number of white blood cells counted [8]. RDTs were performed and reported qualitatively. RDTs and thick smear results were recorded on data collection sheet. Lab coat, gloves, slides, and blood collection equipment were used.

### 2.6. Data Collection Instruments

Data collection forms were used to collect data, and the information was inputted into a computer. The computer was used for safe storage and analysis of the data abstracted.

### 2.7. Data Analysis and Presentation Procedures

Categorical measurements were reported as number and percentage. Quantitative measurements were reported as the mean ± SD (standard deviation). Sensitivity, specificity, positive predictive values, and negative predictive values of RDT in reference to the quantitative method were calculated by using the formulas given in Table 1 and then compared. The statistical analyses were performed by IBM SPSS version 21, a statistical software package.

### 2.8. Inclusion Criteria

Patients included in this manuscript did not receive any antimalarial drug before participating in the study.

### 2.9. Ethical Consideration

This study was revised and approved by a departmental Institutional Review Board committee in the school of Health Sciences of Mount Kenya University, Kigali. Ethical approval was also requested from research committee of the Rukara Health Center. To assure confidentiality, numbers were used as study ID instead of names or hospital ID on patient data collection forms.

## 3. Research Findings and Discussion

### 3.1. Demographic Characteristics of the Study Subjects

The demographic characteristics of the study subjects are given in Table 2. This study included a total of 150 study participants, 87 (58%) females and 63 (42%) males. The mean age of the study participants was 31.6 ± 12.4 with the majority being between 25 and 44 years old and the minority being above 65 years old.

Proportions of malaria by RDT and the quantitative method are given in Table 3. By using rapid diagnostic test (HRP-2), 116 (77.3%) were positive while 33 (22.0%) were negative. In the quantitative method, 67.3% of samples were positive while 32.6% were negative. Sixty four percent (64%) of the tested samples were positive with both RDT and the quantitative method, 3.3% were negative by RDT but positive by the quantitative method, and 19.3% were negative with both RDT and the quantitative method while 13.3% were positive with RDT but negative by the quantitative method.

Proportions of malaria by Giemsa microscopy and the quantitative method are given in Table 4. Not surprisingly, the results of Giemsa microscopy were the same as the results of the quantitative method where both obtained 101 (67.3.0%) positive and 49 (32.6%) negative results. There are no positive results in the quantitative method which got negative in Giemsa microscopy and vice versa.

### 3.2. Presentation of Findings

#### 3.2.1. Sensitivity of RDTs and Giemsa Microscopy in Diagnosis of Malaria

The sensitivity of RDTs (HRP-2) and Giemsa microscopy in diagnosis of malaria is given in Table 5. In this study, the quantitative method was considered as a reference method. Therefore, 64% of the patients who were positive with both RDT and the quantitative method were considered true-positive. The patients who were negative with RDT and positive with the quantitative method were 3.3% and are false-negative results. On other side, 67.3% of positive results by both Giemsa microscopy and the quantitative method were true-positive. As mentioned above, there are no negative results in Giemsa microscopy which got positive in the quantitative method and vice versa. Therefore, false-negative results with Giemsa microscopy are 0.0%. The sensitivity of RDT (HRP-2) was calculated and found to be 95.0%, whereas the sensitivity of Giemsa microscopy was 100%.

#### 3.2.2. Specificity of RDT and Giemsa Microscopy in Diagnosis of Malaria

The specificity of RDTs (HRP-2) and Giemsa microscopy in diagnosis of malaria is given in Table 6. Negative results by both RDT and the quantitative method were 19.3% and are true-negative results. Positive results by RDT but negative by the quantitative method were 13.3% and are false-positive. Again, on the other side, 32.6% of negative results by both Giemsa microscopy and the quantitative method were true-negative, whereas positive results by Giemsa microscopy but negative by the quantitative method were 0.0% and are false-positive. The specificity of RDT (HRP-2) was calculated and found to be 59.2%, whereas the specificity of Giemsa microscopy was 100%.

#### 3.2.3. Positive and Negative Predictive Values of RDT and Giemsa Microscopy

Positive predictive value is the probability that subjects with a positive screening test truly have the disease. Negative predictive value is the probability that subjects with a negative screening test truly do not have the disease. Positive and negative predictive values of RDT (HRP-2) and Giemsa microscopy are calculated as given in Table 7. Negative and positive predictive values of RDT are 85.4% and 82.7%, respectively. These results mean that if tested negative for malaria by RDT (HRP-2), there is 85.4% chance of not having the disease. When tested positive for malaria with RDT (HRP-2), there is a chance of 82.7% of truly having the disease. Negative and positive predictive values of Giemsa microscopy were 100%.

## 4. Discussion

This study showed the sensitivity of RDT (HRP-2) of 95.0% and the specificity of RDT (HRP-2) of 59.2%, whereas the specificity and sensitivity of Giemsa microscopy were 100%. Negative and positive predictive values of RDT were 85.4% and 82.7%, respectively. Negative and positive predictive values of Giemsa microscopy were 100% (Tables 3–7). These parameters were calculated using the formula illustrated in Table 1. Results of Giemsa microscopy were the same as the results of the quantitative method, where both obtained 101 (67.3.0%) positive and 49 (32.6%) negative results (Table 4). This study included a total of 150 study participants where 87 were females and 63 were males, and the mean age of the study participants was 31.6 ± 12.4 (Table 2). The demographic characteristics did not contribute toward the scientific calculations of sensitivity, specificity, and predictive values.

Similar studies were conducted across Africa, Nigeria [9], Angola [10], and Uganda [11]. All these studies in the reviewed literature obtained lower sensitivity, specificity, NPV, and PPV of Giemsa microscopy than ours. This difference is thought to be due to the microscopic qualitative method that was assessed by the similar method. However, in these studies, PCR was used to asses both RDTs and Giemsa microscopy.

Sensitivity of RDTs obtained in this study (95.0%) was too higher than that of the studies conducted by Olusola et al. in Nigeria (62.3%), Cláudia in Angola (60%), and Vincent Batwala et al. in Uganda (91.0%). The specificity of RDTs obtained in this study (59.2%) was lower than that of the studies conducted by Olusola et al. in Nigeria (87.4%) and Cláudia in Angola (94.3%). The NPV and PPV obtained in this study (85.4% and 82.7%) were similar to those obtained by Olusola et al. in Nigeria; however, different results were obtained in the study by Cláudia in Angola (94.8% and 70.7%) and Vincent Batwala et al. in Uganda (95.8% and 88%).

The possible explanation of false-negative RDTs is deletions or mutations within the pfhrp-2 gene or by the prozone effect reported by others [12, 13]. Nevertheless, RDTs were significantly more sensitive than microscopy in most of the reviewed studies, probably corroborating the ability of RDTs to detect parasites below the threshold of microscopy as previously described [14, 15].

There is a great impact of RDT and microscopy in treatment of malaria. In fact, if a patient is positive with RDT at the initial stage without any previous antimalarial drug history, the patient can be treated with antimalarial drugs. On the other hand, if RDT is negative at the initial stage, microscopy is needed in order to confirm the infection because it could be a deleterious mutation. It is also clear that if the quantity of parasites is very low, the false-negative result on microscopy could be due to lack of hands on expertise in microscope reading which is a common problem in capacity building. It is also worth noting that RDT detects genes, whereas microscopy detects parasites; therefore, if a patient revisits the health facility for almost similar symptoms, the RDT may be positive, whereas it can be negative on microscopy. On the other hand, this could be due to reemerging of the disease due to uncompleted dose. Therefore, it is recommended to redo both the tests before retreating the patient again in order to avoid any overdose.

## 5. Conclusions

According to the results of the current study, sensitivity, specificity, and both positive and negative predictive values of Giemsa microscopy (All 100%) are higher than those of histidine-rich protein 2-based rapid diagnostic test for malaria (sensitivity (95%), specificity (59.2%), and PPV (82.7%) and NPV (85.4%). It is worth to say that RDT is an easy and rapid test for malaria diagnosis for quick intervention in treatment. The results obtained in histidine-rich protein 2-based rapid diagnostic test for malaria parasites should be confirmed with tests with high specificity. Further experimental studies should develop the most appropriate type of rapid diagnostic test of malaria diagnosis to be used in combination with Giemsa microscopy. In addition, the sensitivity and specificity of RDT (HRP-2) and Giemsa microscopy should be assessed against molecular biology techniques.

## Figures and Tables

**Table 1:** Formulas that were used in data analysis.

Parameters	Formulas
Sensitivity	Sensitivity=(true‐positive/(true‐positive+false‐negative)) × 100
Specificity	Specificity=(true‐negative/(false‐positive+true‐negative)) × 100
Negative predictive values	NPV=(true‐negative/(true‐negative+false‐negative)) × 100
Positive predictive values	PPV=(true‐positive/(true‐positive+false‐positive)) × 100

**Table 2:** Demographic characteristics of the study participants.

Characteristics	Gender		Total
Age (years)	Females	Males	
<25	32	21	53
25–44	29	26	55
45–64	20	13	33
65+	6	3	9
Total	87 (58%)	63 (42%)	150 (100%)

**Table 3:** Proportions of malaria status by RDT and the quantitative method.

RDT	Quantitative method		Total
	Positive	Negative	
Positive	96 (64%)	20 (13.3%)	116 (77.3%)
Negative	5 (3.3%)	29 (19.3%)	33 (22%)
Total	101 (67.3%)	49 (32.6%)	150

**Table 4:** Proportions of malaria status by microscopy and the quantitative method.

Microscopy	Quantitative method		Total
	Positive	Negative	
Positive	101 (67.3%)	0 (0.0%)	101 (67.3%)
Negative	0 (0.0%)	49 (32.6%)	49
Total	101 (67.3%)	49 (32.6%)	150 (100%)

**Table 5:** Sensitivity of RDTs (HRP-2) and Giemsa microscopy in diagnosis of malaria.

Variables and calculation	Values
RDT	
True-positive	64.0
False-negative	3.3
Sensitivity=(0.64/(0.64+0.033)) × 100	95.0

Microscopy	
True-positive	67.3
False-negative	0.0
Sensitivity=(0.673/(0.673+0.0)) × 100	100

**Table 6:** Specificity of RDTs (HRP-2) and Giemsa microscopy in diagnosis of malaria.

Variables and calculation	Values (%)
RDT	
True-negative	19.3
False-positive	13.3
Specificity=(0.193/(0.193+0.133)) × 100	59.2

Microscopy	
True-negative	32.6
False-positive	0.0
Specificity=(0.326/(0.326+0.0)) × 100	100

**Table 7:** Positive and negative predictive values of RDT and Giemsa microscopy.

Variables and calculation	Values (%)
RDT	
True-negative	19.3
False-negative	3.3
True-positive	64
False-positive	13.3
PPV=(0.64/(0.64+0.133)) × 100	82.7
NPV=(0.193/(0.193+0.033)) × 100	85.4

Microscopy	
True-negative	32.6
False-negative	0.0
True-positive	67.3
False-positive	0.0
PPV=(0.673/(0.673+0.0)) × 100	100
NPV=(0.326/(0.326+0.0)) × 100	100

PPV: positive predictive values. NPV: negative predictive values.

## Data Availability

All materials and data are available.
